# *In silico* Interrogation of Insect Central Complex Suggests Computational Roles for the Ellipsoid Body in Spatial Navigation

**DOI:** 10.3389/fnbeh.2017.00142

**Published:** 2017-08-03

**Authors:** Vincenzo G. Fiore, Benjamin Kottler, Xiaosi Gu, Frank Hirth

**Affiliations:** ^1^School of Behavioral and Brain Sciences, University of Texas at Dallas Dallas, TX, United States; ^2^Department of Basic & Clinical Neuroscience, Institute of Psychiatry, Psychology & Neuroscience, King's College London London, United Kingdom

**Keywords:** insect brain, central complex, ellipsoid body, lateral accessory lobes, computational model, spatial navigation, cognitive map

## Abstract

The central complex in the insect brain is a composite of midline neuropils involved in processing sensory cues and mediating behavioral outputs to orchestrate spatial navigation. Despite recent advances, however, the neural mechanisms underlying sensory integration and motor action selections have remained largely elusive. In particular, it is not yet understood how the central complex exploits sensory inputs to realize motor functions associated with spatial navigation. Here we report an *in silico* interrogation of central complex-mediated spatial navigation with a special emphasis on the ellipsoid body. Based on known connectivity and function, we developed a computational model to test how the local connectome of the central complex can mediate sensorimotor integration to guide different forms of behavioral outputs. Our simulations show integration of multiple sensory sources can be effectively performed in the ellipsoid body. This processed information is used to trigger continuous sequences of action selections resulting in self-motion, obstacle avoidance and the navigation of simulated environments of varying complexity. The motor responses to perceived sensory stimuli can be stored in the neural structure of the central complex to simulate navigation relying on a collective of guidance cues, akin to sensory-driven innate or habitual behaviors. By comparing behaviors under different conditions of accessible sources of input information, we show the simulated insect computes visual inputs and body posture to estimate its position in space. Finally, we tested whether the local connectome of the central complex might also allow the flexibility required to recall an intentional behavioral sequence, among different courses of actions. Our simulations suggest that the central complex can encode combined representations of motor and spatial information to pursue a goal and thus successfully guide orientation behavior. Together, the observed computational features identify central complex circuitry, and especially the ellipsoid body, as a key neural correlate involved in spatial navigation.

## Introduction

Ambulatory animals are constantly subject to changing stimuli. These include external sensory stimuli, such as light, temperature or food locations; and internal stimuli, such as body posture, position in space, thirst or hunger. Efficient mechanisms to identify, consolidate and recall information and appropriate motor actions are essential for the animal's ability to respond to the external stimuli, avoid obstacles, move away from potential threats or approach hedonic rewards. Accordingly, hunters, foragers or harvesters have evolved neural mechanisms that exploit the integration of changing internal and external stimuli to trigger action sequences in order to drive both goal-driven behaviors and reactive sensory-driven habits. The selection of appropriate motor commands allows the animal to change position in space or to interact with elements in the environment. This self-motion information is then computed jointly with new incoming sensory stimuli to consolidate memory of experienced action-outcome contingencies, in association with allocentric and egocentric representations. Eventually, the association of outcomes with a representation of sensory stimuli, body posture, and actions result in a mental map (Tolman, 1932; Collett et al., 2013), which guides adaptive behavior and is essential for intentional spatial navigation.

Like all ambulatory animals, insects express behaviors that result in intentional spatial navigation. For instance, complex visual features (Neuser et al., 2008; Ofstad et al., 2011) or antennal mechanosensations (Ritzmann et al., 2008; Varga et al., 2017) perceived whilst exploring an arena, can be learned and stored to subsequently recall an action (Neuser et al., 2008; Ofstad et al., 2011). However, it has remained contentious whether insects use spatial representations to guide their navigation (e.g., Cheeseman et al., 2014; Cheung et al., 2014) or rather their orientation behavior relies on a collective of guidance cues (Cruse and Wehner, 2011; Collett et al., 2013) that include neural correlates of head direction (e.g., Varga and Ritzmann, 2016), celestial compass cues (e.g., el Jundi et al., 2015) and configurations of visual stimuli in view-based panoramas (e.g., see Zeil, 2012; Seelig and Jayaraman, 2015; Weir and Dickinson, 2015). A key neural correlate involved in processing these guidance cues and mediating behavioral outputs resulting in spatial navigation is the central complex (CX) (Strausfeld and Hirth, 2013; Pfeiffer and Homberg, 2014; Turner-Evans and Jayaraman, 2016; Webb and Wystrach, 2016).

**Figure 1:**
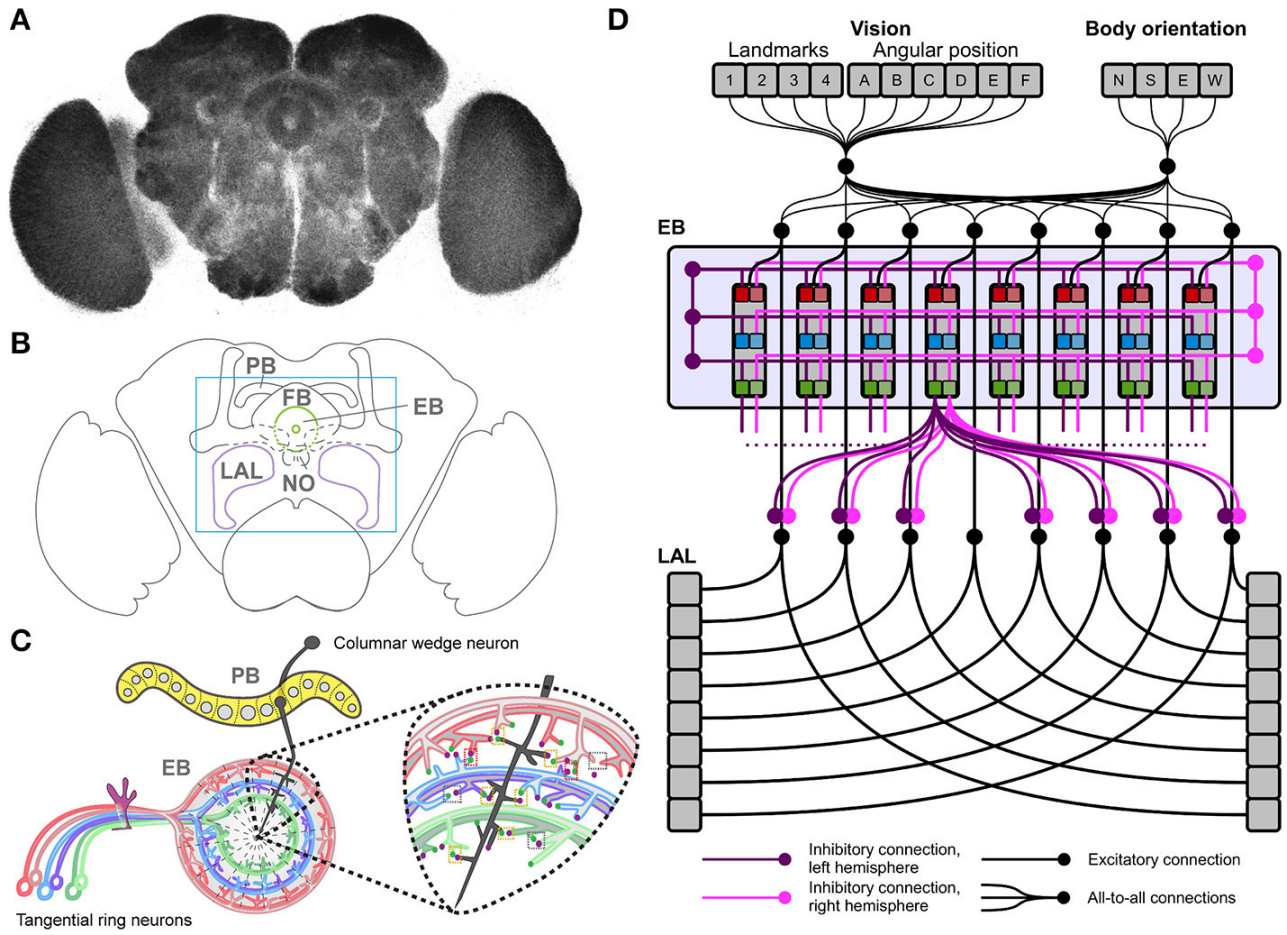
The *Drosophila* central complex and derived architecture of the computational model. **(A)** Confocal image (color inverted) of a dissected adult *Drosophila* brain immuno-labeled with an antibody that specifically recognizes synaptic terminals. **(B)** Cartoon of adult *Drosophila* brain showing central complex neuropils (PB, protocerebral bridge; FB, fan-shaped body; EB, ellipsoid body; NO, noduli; LAL, lateral accessory lobes—mushroom bodies are shown for orientation); box indicates enlarged region in C. **(C)** Schematic summary of PB and EB to show key assumptions of computational model: columnar wedge neurons project to specific EB wedges (here shown for one in black), tangential ring neurons project in a subtype and layer-specific manner into EB ring (colour coded); the model assumes synaptic connections between both neuron types (see enlarged area for one wedge). **(D)** Architecture of the neural model, replicating the local connectome of the central complex in the connections among modules.

The CX is a central brain structure composed of midline neuropils comprising the protocerebral bridge (PB), the fan-shaped body (FB), the ellipsoid body (EB), the noduli and the lateral accessory lobes (LAL) (Figures 1A,B). Histological (Williams, 1975; Hanesch et al., 1989), immunocytochemical (Hanesch et al., 1989; Renn et al., 1999; Young and Armstrong, 2010; Kahsai and Winther, 2011; Boyan and Liu, 2016), and clonal analyses (Ito and Awasaki, 2008; Ito et al., 2013; Lin et al., 2013; Wolff et al., 2015) reveal the CX organization as a modular system of neuronal layers and columns (Figure 1C). Columnar neurons leading to and from the CX connect all its component neuropils (Ito and Awasaki, 2008; Lin et al., 2013; Wolff et al., 2015), which are themselves subdivided into modules. These modules encode spatial information about sensory events (Heinze and Homberg, 2007; Sakura et al., 2008; Heinze and Reppert, 2011; Seelig and Jayaraman, 2013). As shown for the EB, tangential layers of neural processes intersect columnar projections and modulate spatial representations of sensory events across modules (Vitzthum et al., 2002; Heinze and Homberg, 2007; Sakura et al., 2008; Heinze and Reppert, 2011; Rosner and Homberg, 2013). Specific sensory inputs (afferents signaling “**what**” is perceived and its features) are mapped across modules (afferents signaling “**where**” the stimulus is located with respect to the body and the environment), each representing a segment of sensory space (Strausfeld, 2012). These representations are relayed to the EB, which weights them according to input salience and strength of connectivity. Finally, the strength of connectivity can be modulated by dopamine-related learning processes (Waddell, 2013), so that the EB effectively integrates current and previous information about all its incoming sensory inputs. In turn, the EB processes its incoming input to release the inhibition of appropriate premotor programs in the LAL, selecting actions in response to the computed sensory stimuli (Fiore et al., 2015; Kottler et al., 2017).

This proposed model of CX functionality identifies the EB as a key node in mediating sensorimotor integration and action selection for reactive stimulus-responses and goal-directed behavior, thus driving purposeful spatial navigation. In support of this notion, recent studies identified columnar neurons that project from PB to EB where they form wedge-specific arborisations that together cover all layers and modules of the EB, and thus all segments of sensory space represented in the EB (Seelig and Jayaraman, 2015; Wolff et al., 2015; Omoto et al., 2017; Figure 1D). These studies propose that visual cues and their positions are represented in the EB relative to the animal's heading. If correct, this hypothesis suggests that columnar wedge-neuron activity encodes an internal compass that combines visual landmarks with self-generated (idiothetic) cues (Heinze, 2015). Despite these advances, the neural mechanisms underlying sensory integration and motor action selections have remained largely elusive. In particular, it is not clear how the CX exploits sensory inputs and encoded head and body orientation to realize motor functions associated with spatial navigation.

We previously proposed that all of these functions may rely on computational processes that can also be found in the vertebrate basal ganglia (Strausfeld and Hirth, 2013). In particular, transient winner-take-all competitions (Rabinovich et al., 2001; Afraimovich et al., 2008) may be a common solution across species for the essential functions of sensory noise suppression, detection and selection of salient inputs weighed by previous experience and sensorimotor integration (Fiore et al., 2015). Here we examine how these functions can be implemented by the CX, as a simulated insect is required to navigate arenas of increasing complexity, to reach two target regions, whilst avoiding obstacles. To solve its task, the simulated insect relies on a heterogeneous set of sensory information about body orientation and visual landmarks, organized in an egocentric representation. These inputs are processed in a bio-constrained neural model (i.e., whose structure is constrained by known neuroanatomy) simulating the neural activity of the EB and LAL as parts of the CX. Our model relies on evidence-based assumptions (Ito et al., 2013; Lin et al., 2013; Seelig and Jayaraman, 2015; Wolff et al., 2015; Kottler et al., 2017) that a somatotopic columnar input organization and lateral inhibitions can generate transient winner-take-all competitions. The behavior of the simulated insect shows the activity in the ellipsoid body can integrate and encode inputs from different sensory sources, and successfully rely on visual information and body orientation to correctly estimate its position in space.

## Materials and methods

### Neural architecture and computational features of the model

We developed a neural model based on an architecture that replicates known connectivity of the CX, focusing on afferent and efferent EB projections (Figure 1D). The model relies on two core features, a loop architecture between EB and LAL, and lateral inhibition among tangential EB ring neurons, both of which are supported by clonal, immunocytological, and functional analyses (Hanesch et al., 1989; Kahsai and Winther, 2011; Lin et al., 2013; Seelig and Jayaraman, 2015; Wolff et al., 2015; Kottler et al., 2017).

The model simulates activity in the modules of EB and LAL in a continuous-time differential equation termed “leaky integrator,” which is used to simulate the mean-field activity of an entire pool of neurons (Deco et al., 2008):

(1) $$\begin{array}{l} \tau_{g}{\dot{u}}_{j}=-u_{j}+b_{j}+\underset{i}{\sum }w_{ji}y_{i} \end{array}$$

(2) $$\begin{array}{l} y_{j}={\left[{\text{tanh}(\text{u}}_{\text{j}})\right]}^{+} \end{array}$$

Equation (1) defines the activation potential of a generic unit *j* and Equation (2) defines the final activation of the unit in a positive saturation transfer function. $\underset{i}{\sum }w_{ji}y_{i}$ represents the overall input reaching unit *j* from all units *i* and *w*_*ji*_ represents the connection weight between an input unit *i* and a target unit *j*. Finally, a bias *b*_*j*_ represents the basal activity of the unit *j*. The value of this constant is equal to 0 under all conditions, with the exception of those simulating either deactivation or overactivation of the EB, when the value is set < −0.5 for deactivation and >0.5 for overactivation, for all EB units.

The neural architecture of the model was based on the projections of columnar neurons which divide the CX into 8 units/columns per hemisphere; and tangential neurons which in the case of the EB project in a subtype specific manner to generate 3 layers of the EB neuropil (Strausfeld and Hirth, 2013; Pfeiffer and Homberg, 2014; Turner-Evans and Jayaraman, 2016; Webb and Wystrach, 2016). Hence our model consisted of 48 units for the EB (16 modules, also called wegdes, each subdivided into 3 layers). Furthermore, based on neuroanatomical and functional studies (Williams, 1975; Hanesch et al., 1989; Namiki and Kanzaki, 2016) we assumed 16 units for the LAL (one unit per LAL segment or module, grouped in 8 units per hemisphere). Sensory information was organized in vectors, where elements represented landmark features and their allocentric position, body orientation of the simulated insect and its position in space (see subsections below). All elements in the input sources were connected with all modules in both EB and LAL, allowing for the integration of heterogeneous sensory information. Tangential ring neuron projections (Fiore et al., 2015; Kottler et al., 2017) were modeled following a computationally parsimonious assumption, which assumed symmetric lateral inhibitions within each layer and among all layers of the EB, per hemisphere (Figure 1D). Lateral inhibitions among layers and modules/wedges resulted in the competition among inputs and the subsequent selection of the strongest signal among competitors (see also: Kottler et al., 2017). In turn, this competition resulted in a transient winner-takes-all functionality, which replicated the structurally stable dynamics reported for the EB (Seelig and Jayaraman, 2015). This selection process carried out in the EB was biased by the weights of the connections streaming sensory information toward the EB itself. Thus, the behavior of the simulated insect ultimately depended on the configuration of the parameters representing the weights *w*_*ji*_ between sensory inputs and EB.

In the model, information processed in the EB (sensory integration and selection) was then streamed to the LAL via inhibitory connections (Fiore et al., 2015; Wolff et al., 2015), conveying EB-mediated selections into premotor outputs. The topology of the inhibitory connections linking EB–LAL has been only partially described in the literature. Thus, we completed the model connectivity relying again on a computationally parsimonious assumption, where all EB modules exerted an off-center gating function toward the two separate layers of the LAL (Figure 1D). Layers in the LAL encode premotor commands, which provide essential feedback to the EB in terms of self-motion information (Namiki and Kanzaki, 2016), besides triggering motor selections. In the simulated agent, LAL self-motion information is conveyed via parallel connectivity from both layers in the LAL toward all layers in the EB, completing the EB-LAL-EB loop. Motor selections were modeled in a simple correlation between activity in the eight modules of the two LAL layers and the execution of basic motor commands. For the spatial navigation task, we mapped the actions *move forward, turn right* (clock-wise) and *turn left* (counter-clockwise) to the activity of three arbitrary modules (per hemisphere). Activity in the remaining five modules (per hemisphere) was used to trigger a series of actions (e.g., grooming, eating, standing still etc.,) that did not produce any change in terms of the position of the simulated insect in the arena or its body orientation. Although these actions were not relevant in terms of spatial navigation, they were part of the transient competition for motor commands and could be selected in response to any combination of perceived sensory stimuli. Thus, the described configuration of motor commands was meant to illustrate how sensory integration can trigger a sequence of actions—it does not represent the entire repertoire of actions that can be performed by an insect. The specifications of our model are well supported by experimental evidence that identifies key roles for the EB in sensorimotor integration and goal-directed behavioral output (e.g., Martin et al., 1999; Heinze and Homberg, 2007; Neuser et al., 2008; Lebestky et al., 2009; Kong et al., 2010; Ofstad et al., 2011; Seelig and Jayaraman, 2015; Kottler et al., 2017) which are essential for spatial navigation.

### Simulated environment

**Figure 2:**
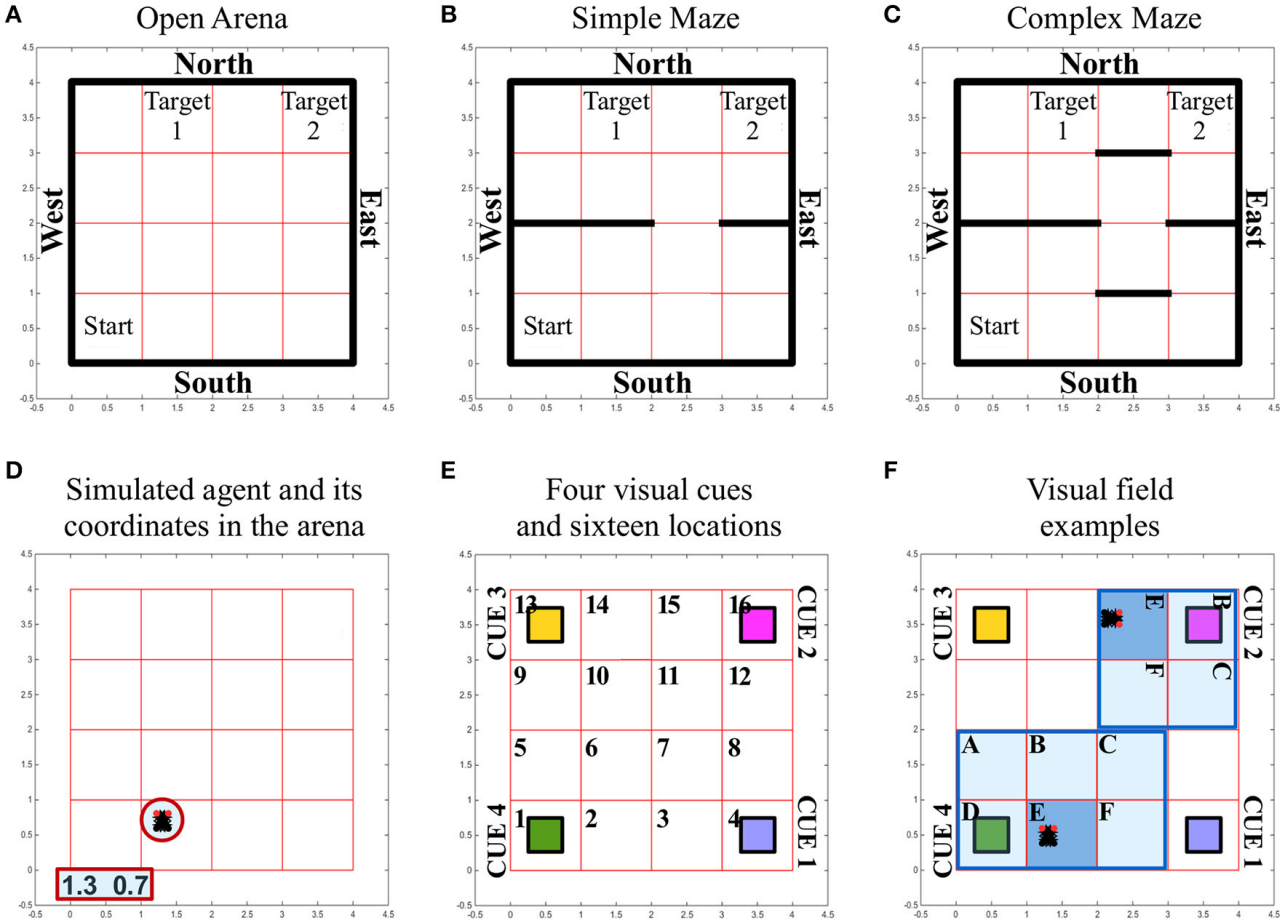
Simulated environment and insect specifics. **(A–C)** Different arenas and corresponding boundaries for the spatial navigation tasks of the simulated insect: open arena **(A)**, simple maze **(B)** and complex maze **(C)**. Under all conditions, arenas are composed of 40 × 40 locations that can be occupied by the simulated insect while it navigates the virtual space. The simulated insect starts each trial in one location, randomly selected among the 100 locations characterizing the south-west sector of the arena, marked as “Start.” The simulated insect can navigate the space freely for a limited time of 30 s of simulated time. The simulation is considered successful if the insect reaches any of the locations defining the target areas (marked as “Target 1” and “Target 2”). At each step of the simulation, the insect can select an action that allows it to leave the location it occupies and move into a new location north, south, east or west. These movements are only prevented if the location occupied by the insect is close to a *wall* (marked in black), in which case the action is not executed and the agent keeps its previous position. **(D)** The simulated insect is graphically represented with black segment for its body and two small red circles for its eyes, to indicate the front of the simulated insect. At any time the location occupied by the insect is reported in terms of coordinates, outside the arena. **(E)** Grid organization and perceived landmarks in the arenas. Under all conditions, the arena is divided into 16 sectors composed of 10 × 10 locations. Four, differently colored, visual cues or landmarks are located at the four corners under all conditions. **(F)** Illustration of the way the visual field adapts depending on the position and body orientation of the simulated insect. The agent can perceive a visual landmark, activating the corresponding visual input to a value of 1, only if this is found in the sector the insect is occupying (E, dark blue sector of the visual field). If the landmark is found in a sector on the front-left (A), front (B), front-right (C), left (D), or right (F) of the simulated insect (pale blue sectors of the visual field), the corresponding visual input signal the presence of the cue with a value of 0.5. Angular position units encode, with a 0/1 activity, an egocentric representation of the position of the landmarks with respect to the body of the simulated insect (one unit per each sector in the visual field). In panel (F), we illustrate two arbitrary examples of the egocentric landmark representation in terms of sensory input. In these examples, angular position units would signal the presence of a generic landmark on the left of the body in one case (cue 4 in sector D of the visual field) or in front of the body in the other case (cue 2 in sector B of the visual field). See also related Supplementary Videos.

We tested the navigation behavior of an artificial insect in a simulated environment that allowed manipulation of its complexity and of the source of sensory information available for the orientation of the simulated insect. Three different environments or arenas were used to limit the movements of the simulated insect and to set obstacles between a starting position and a target area. Independent of the complexity of the simulated environment, all arenas/environments were composed of 1600 distinct locations (40 × 40). In each of the different arenas, external *walls* defined the overall number of locations. The starting position of the simulated insect was randomly selected out of 100 locations (10 × 10) in the southwest of the arena. Two target areas were defined as squares of 10 × 10 locations, both of which were placed in the northern part of the arena, thus leading to a considerable distance between the artificial insect's starting location and the target area it had to reach (Figure 2A).

The complexity of the simulated environment was modified by introducing three conditions, characterized by an increasing number of obstacles that had to be circumnavigated in order to reach the target areas. Thus, in the environment termed “***open arena***” (Figure 2A), the simulated insect was able to change location by freely moving North, East, South or West. If the simulated insect reached any of the arena walls and tried to execute a command to move further, the command was ignored and the agent remained in its position. In the environment termed “***simple maze***” (Figure 2B), the arena presented internal obstacles as additional walls. These walls divided the arena in half with the exception of a narrow passage of one sector width (equal to 10 locations), thereby limiting the ability of the simulated insect to cross from South to North and vice versa. In the environment termed “***complex maze***” (Figure 2C), additional obstacles were introduced to further limit the movements of the agent, thereby forcing the simulated agent to execute a series of at least five well-timed turns to be able to reach a target area.

### Simulated sensory information

The simulated insect relied on two sources of sensory information that were made available either in combination or alone.

#### Self-motion

To illustrate the anterior-posterior orientation in relation to the arena's polarity (N-E-S-W), the simulated insect was graphically represented in videos and images with two red circles for the eyes (e.g., see Figure 2D and Supplementary Videos). This “body orientation” was encoded in a four dimensional vector characterized by a binary 0/1 activity. The activity of this vector changed with the execution of turning behavior and it was propagated toward the CX. Jointly with the vector of activity recorded in the LAL and determining motor selections in the simulated agent, these two signals provided the agent information about self-generated motion and posture.

#### Vision

Any movement resulting in changing the position of the simulated insect or its body orientation could result in the modification of its visual field. To simulate this dynamic change, we first divided the arenas into 16 sectors of 10 × 10 locations (Figure 2E). Second, we defined the visual field as covering the sectors in front and on the side of the insect, in a putative 180° forward-facing arc (Figure 2F). If any of the visual cues or landmarks located at the four corners of the arena entered the visual field of the simulated insect, its presence was encoded in two signals, representing objects in terms of “*what*” is perceived, and its angular position, an egocentric “*where*” they are perceived. We simplified the neural representation of the unique visual features of each landmark (i.e., color) by providing a different *visual unit* per each landmark, in a localistic representation. The activity of a visual unit was set to 1 if the corresponding landmark was located in the same sector occupied by the simulated insect. The same value was set to 0.5 when a cue was located anywhere else in the visual field, therefore responding to the presence of a landmark independently of its position. To spatially represent and differentiate between landmarks located, for instance, on the left rather than on the right of the body, a second source of visual input was conveyed via six *angular position units*. These units encoded, with a binary 0/1 activity, an egocentric representation of the position of the landmarks with respect to the body of the simulated insect (i.e., each unit was active to signal the presence of a landmark located, respectively: left, front-left, front, front-right, right, or in the same sector of the simulated insect body; Figure 2F). In contrast to visual units, angular units could not differentiate among landmarks, so that the agent needed to integrate both sources of visual information in order to determine which landmark was visible and where.

#### Putative desired outcomes

We explored whether the modeled CX could store multiple sequences of actions at the same time, and recall the correct one, depending on a desired outcome. Therefore, we provided the modeled CX with two “biases” simulating a physiological assessment of the body status (e.g., representing *hunger* and *thirst*). Under this condition, termed “***intentional spatial navigation***,” each of the two target areas was assumed to satisfy only one of the two desired outcomes: target 1 was associated with bias 1 and target 2 was associated with bias 2. The biases were activated in sequence and maintained active until the end of the trial time or until the appropriate target area was found.

#### Parameter estimation

The selection process eventually carried out in the EB is biased by the weights of the connections streaming sensory information toward the EB itself. Thus, the behavior of the simulated insect ultimately depends on the configuration of the parameters representing the weights *w*_*ji*_ between sensory inputs and EB. In a real-life experiment, an insect would randomly explore the physical equivalent of the proposed simulated arenas, eventually reaching one of the target areas. In presence of unexpected positive outcomes (e.g., food or water), reinforcement learning processes would occur (Sutton and Barto, 1998), thereby altering the connection weights between sensory regions and the EB (Waddell, 2010, 2013). In the long run this process results in instrumental conditioning, effectively generating and storing motor responses to perceived stimuli in the connection weights that bias the selection process in the EB. For the time being, we did not simulate fast dopamine burst firings in our model, which are essential in regulating the learning process (Schultz, 2002; Waddell, 2010, 2013). Therefore, we tested the simulated insect under the theoretical assumption that it had already completed its training and formed its stimulus-response associations. This assumption entails there are configurations of connection weights *w*_*ji*_ that allow the simulated insect to exploit the sensory information and recall a path of motor responses to navigate the arena. We looked for such configurations of parameters relying on a Monte Carlo method for parameter estimation and tested the simulated insect in two million randomly sampled configurations, or behavioral phenotypes, per each condition.

#### Software

The model, Monte Carlo parameter estimation and simulated interaction between environment and agent were developed and run in MatLab in *ad hoc* libraries.

## Results

We exposed the simulated insect to three different arenas of increasing complexity. The sensory inputs conveyed information about visual landmarks and self-motion that changed dynamically, depending on body orientation of the simulated insect and its location in the arena. We hypothesized the simulated insect can rely on the accessible information as guidance cues for both reactive sensory-driven and intentional spatial navigation (Table 1). Our model explored two key assumptions:

**Table 1:** Successful behaviors over attempts ratios.

**Available Input sources**	**Open arena**	**Simple maze**	**Complex maze**
Vision +	Target 1–6,950:10^6^	Target 1–67:10^6^	Target 1–0.5:10^6^
Body orientation	Target 2–1,335:10^6^	Target 2–175:10^6^	Target 2–18:10^6^
Vision	Target 1–0:10^6^	Target 1–0:10^6^	Target 1–0:10^6^
	Target 2–0:10^6^	Target 2–0:10^6^	Target 2–0:10^6^
Body orientation	Target 1–0:10^6^	Target 1–0:10^6^	Target 1–0:10^6^
	Target 2–0:10^6^	Target 2–0:10^6^	Target 2–0:10^6^

### Columnar wedge neurons integrate visual landmarks with idiothetic cues

Visual information available to the insect (Figure 2E) was simulated by two signals, encoding the perceived object features and egocentric location. Each of the four *visual units* was used to respond to the presence of a specific landmark in the visual field (Figure 2F), independently of its egocentric position. Information about the egocentric position of perceived landmarks was conveyed via six (for landmarks located in a sector on the left, right, front-left, front-right or front of the insect body, or in the same sector of the agent) *angular position units*, which could not differentiate among landmarks. Our simulated agent had to integrate both types of visual information jointly with body orientation and self-motion feed-back information to solve the task and accomplish purposeful navigation. This process of sensory integration simulated information encoded in the real PB->EB columnar wedge neuron activity (Seelig and Jayaraman, 2015) that combines visual landmarks with self-generated cues (Heinze, 2015).

### Tangential ring neurons mediate motor action selection

In addition to columnar wedge neurons, we included tangential ring neurons of the EB into the model architecture. Based on lineage analyses revealing their terminal arborisations (Ito et al., 2013; Lin et al., 2013; Wolff et al., 2015), we simulated three ring neuron subtypes and layers (R1, R2/4, and R3), each divided into 16 wedges, and implemented connections between columnar wedge and tangential ring neurons in a layer- and wedge-specific pattern in the EB ring neuropil (Figures 1C,D). Given the lack of information about EB internal organization or hierarchy among layers, we implemented symmetric lateral inhibitions among ring neurons (Fiore et al., 2015; Kottler et al., 2017). These lateral inhibitions established a competition among incoming inputs, which resulted in the transient selection of salient stimuli (Rabinovich et al., 2001; Afraimovich et al., 2008). This transient winner-takes-all functionality was consistent with the dynamics reported for the EB (e.g., Seelig and Jayaraman, 2015; Kottler et al., 2017). Finally, EB-mediated selections were conveyed into premotor outputs via the inhibitory gating exerted by the EB toward the LAL. The simulated LAL encoded premotor commands that triggered motor activity and provided essential feedback of self-motion information (Namiki and Kanzaki, 2016).

### *In silico* interrogation of EB-mediated spatial navigation

We applied these assumptions in our model and utilized the *Monte Carlo* method to compute two million randomly generated patterns of parameters or behavioral phenotypes. This allowed us to investigate configurations of parameters that might have enabled the simulated insect to reach the target areas by responding to incoming sensory stimuli, under each condition. We used this sampling to determine a ratio that captures the number of successful navigations per million attempts, per condition. The resulting value thus provides a proxy for the duration of a putative exploration required to successfully find a target area in the arenas. High ratio values correspond to frequent discovery of successful configurations of parameters, or alternative successful paths for the simulated insect, therefore implying a short exploration time. As expected, the resulting ratios suggested that successful spatial navigation is dependent on the complexity of the arena explored and the quality and combination of available sensory sources (Table 1).

### Sensory-driven navigation

**Figure 3:**
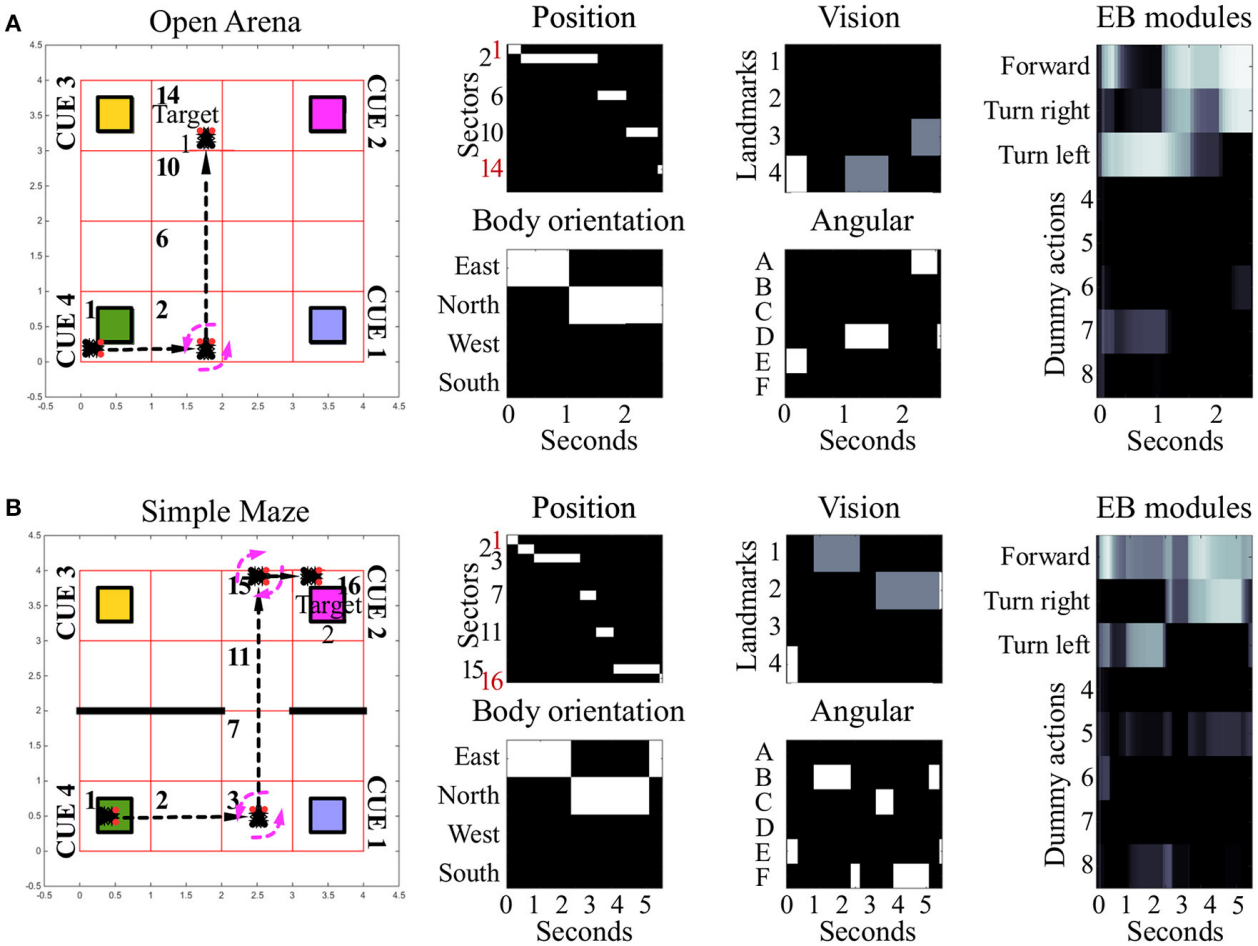
Examples of spatial navigation under the conditions *open arena* and *simple maze*. **(A)** Example of navigation in the open arena, in which the simulated insect walks from its starting position toward the target area 1. In this example the simulated insect starts from sector 1, then turns left whilst in sector 2, and proceeds forward crossing sectors 6 and 10, to reach the target in sector 14 (see also related Supplementary Videos 1, 2). **(B)** Example of navigation in the simple maze from starting position toward target area 2. Under this condition, the simulated insect has to move east from its starting position, crossing sector 2, to turn left whilst in sector 3. Then it has to move north, across sectors 7 and 11, before turning right, whilst in sector 15, and complete the path by reaching sector 16 (see also related Supplementary Videos 3, 4). The path of the simulated insect is also represented with a black and white heat-map under the label “Position.” This heat-map allows to track the position of the simulated insect at any step of the simulation, where a binary 0/1 activity encodes the presence of the agent in any of the sectors of the arenas (16 units, one per sector). The inputs reaching the EB are reported under the labels: “*body orientation*” (4 units, one per possible direction, binary 0/1 activity), “*vision*” (4 units, one per landmark or visual cue, responding with fixed values of 0, 0.5, or 1, depending on the distance of the landmark), and “*angular*” (6 units, one per egocentric position of any landmark in the visual field, responding with a binary 0/1 activity). Finally, the black and white heat-map, labeled “EB modules,” represents the activity of the modules in the EB in a single hemisphere. This heat-map responds with continuous values between 0 and 1 and encodes the average activity across the three layers of the EB ring neuropil. In the simulated EB, the competition among modules triggers the selection of one among eight possible actions via gating of LAL premotor activity. The only actions resulting in changes of the simulated insect position or body orientation are encoded in the first three modules as follows: move forward (module 1), turn left (module 2) and turn right (module 3). The other five actions remain part of the competition in the EB, but represent motor activities (e.g., grooming, eating, standing still etc.,) which do not result in movement in the arena and thus do not change spatial navigation behavior.

Under the condition termed as “open arena” the simulated insect was able to change location by freely moving North, South, East or West. Therefore, the optimal behavior, marking the shortest path between starting location and target area, required only one turn, to the left (Figure 3A, see also Supplementary Videos 1, 2). Nonetheless, many other suboptimal behaviors (longer than the shortest path) were still successful, allowing the simulated insect to reach the target area within the time limit, despite the fact they relied on multiple turns and unnecessarily long paths. Information provided by vision and self-motion allowed the algorithm to find 6950 successful behavioral phenotypes per million attempts to reach target area 1 and 1,335 per million attempts to reach target area 2. Neither visual information nor self-motion information, considered separately, proved to be sufficient to solve the task.

**Figure 4:**
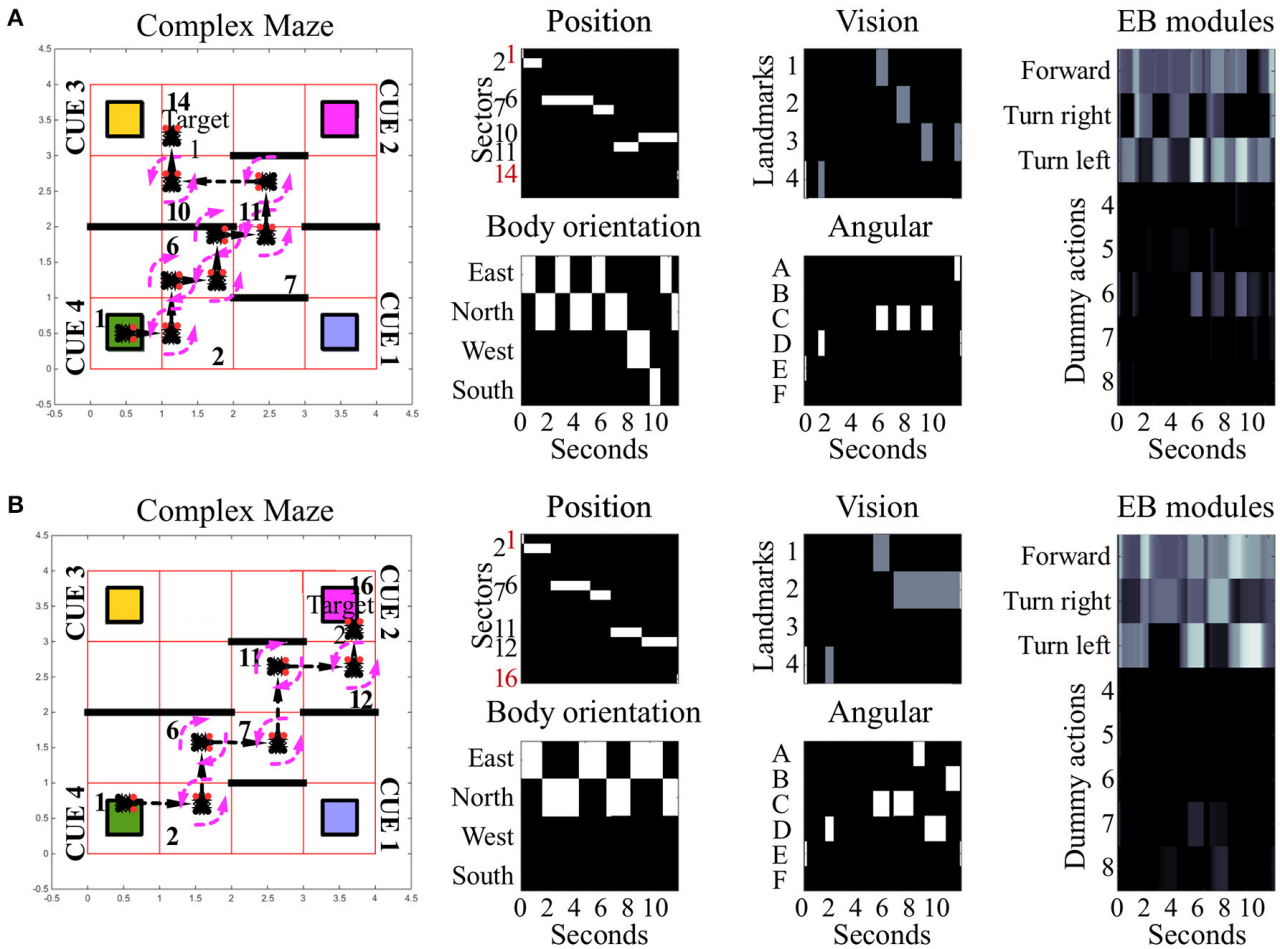
Examples of spatial navigation under the complex maze condition. Both panels illustrate examples of navigation under the complex maze condition (see also related Supplementary Videos 5, 6). **(A)** Motor behavior of the simulated insect, from its starting position toward target area 1. To avoid the obstacles in the arena, the insect turns 7 times and crosses 7 sectors. **(B)** Motor behavior displayed by the simulated insect to reach target area 2. In comparison to **(A)**, the insect reaches its target crossing 5 sectors in total. Under this maze condition, obstacle avoidance requires 5 turns in both versions of the task with the example in **(A)** reporting sub-optimal behavior. In both examples, the path of the simulated insect can be tracked in a black and white heat-map under the label “Position.” This heat-map continuously records the position of the agent during the simulation time, responding with a binary 0/1 activity to the presence of the agent in any of the sectors of the arenas (16 units, one per sector). The inputs reaching the EB are reported under the labels: “*body orientation*” (4 units, one per possible direction, binary 0/1 activity), “*vision*” (4 units, one per landmark or visual cue, responding with fixed values of 0, 0.5, or 1, depending on the distance of the landmark), and “*angular*” (6 units, one per egocentric position of any landmark in the visual field, responding with a binary 0/1 activity). Finally the black and white heat-map, labeled “EB modules,” represents the activity of the modules in the EB in a single hemisphere. This heat-map responds with continuous values between 0 and 1 and encodes the average activity across the three layers. In the simulated EB, the competition among modules triggers the selection of one among eight possible actions via gating of LAL premotor activity. Activity in the LAL triggers the execution of 8 different actions which can all be selected via the gating function exerted by the EB. Three of these actions result in changes of body orientation or position of the simulated insect and are encoded in the first three modules: move forward (module 1), turn left (module 2) and turn right (module 3). The remaining five actions putatively represent motor activities, such as grooming, eating or standing still which are not graphically represented in terms of behavioral execution.

In the “simple maze” arena, the internal walls divided the arena in half, with a narrow passage limiting the movements of the simulated insect from its starting point toward both targets. The arena termed “complex maze” introduced further internal walls that required the simulated insect to execute multiple turns to reach the target areas. Independently of the target area, optimal behavior required two turns in the simple maze (Figure 3B, see also Supplementary Videos 3, 4) and five turns in the complex maze (Figure 4, see also Supplementary Videos 5, 6). These limits significantly reduced the number of suboptimal behaviors that could successfully solve the task, thus diminishing the chances the search algorithm would be able to find solutions via random sampling. Nonetheless, the search algorithm found several successful configurations of parameters under both simple maze and complex maze conditions (Table 1). These allowed the simulated insect to use visual cues and body orientation information to trigger the appropriate sequence of actions, resulting in turns and navigation behavior to avoid the obstacles and reach the target areas. Neither of the input sources, considered alone, endowed the search algorithm with a successful behavior (Table 1).

**Figure 5:**
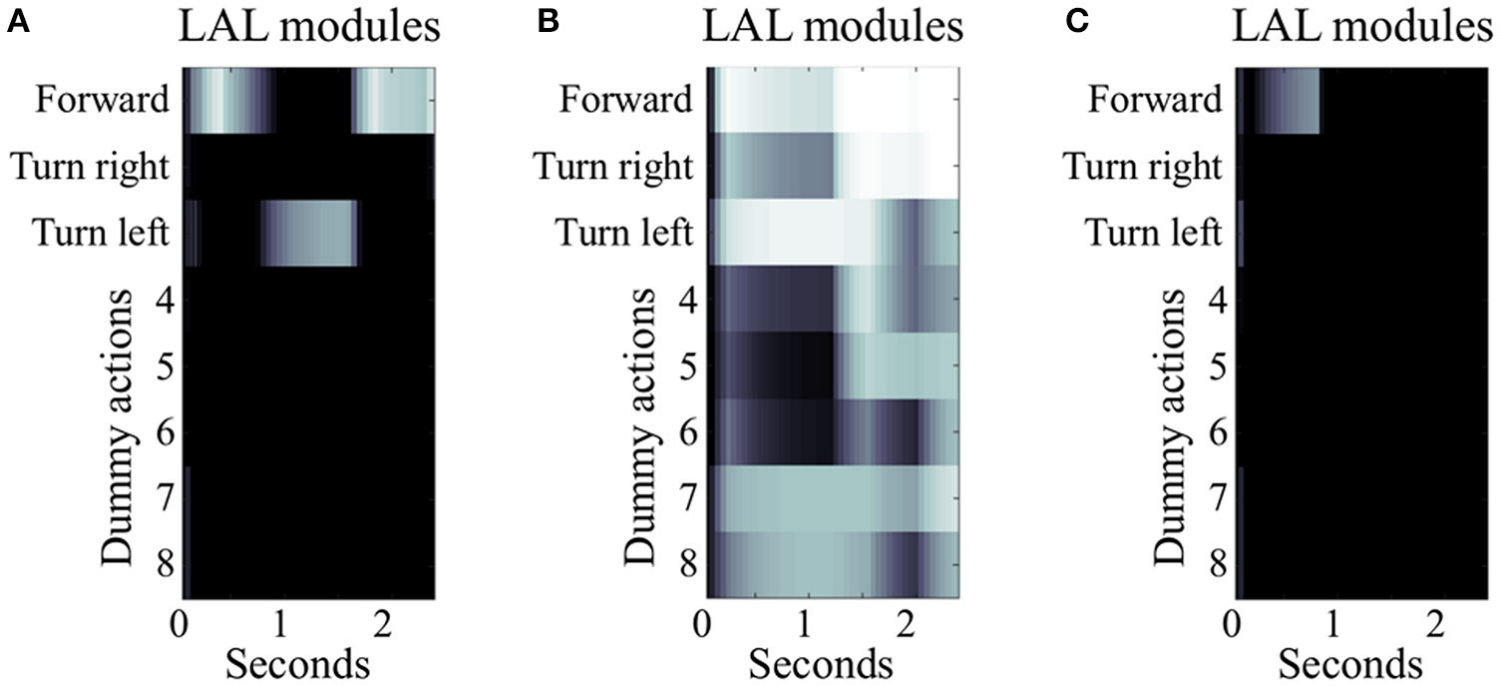
Simulated LAL under different conditions of EB manipulation. The three panels illustrate 2.5 s of simulated activity recorded in the LAL, single hemisphere, under different condition of EB manipulation. **(A)** Under control condition, the heatmap shows differentiated activity in the modules of the LAL, with transient selections due to the gating inhibition performed by the EB. In this example, the simulated insect successfully moves forward, turns left, and moves forward again until it reaches target area 1. **(B)** Under the condition of EB deactivation, the gating function is diminished leading to general overactivation of several modules in the LAL and loss of differentiation in the signal. In this example, the simulated insect is able to perform the initial motor activity, but it stops when the actions “forward” and “turn right” become both strongly active. **(C)** Under the condition of EB overactivation, the gating function exerted by EB on LAL is potentiated. In this example, LAL is only able to trigger one action (move “forward”) at the beginning of the simulation, when the visual input is strong enough to overcome the inhibition provided by the EB. As soon as the simulated insect changes its location and the visual input is not accessible, the remaining sensory stimuli are unable to drive any premotor response, leaving the simulated insect motionless.

Under all conditions of environment complexity, these tests show that the simulated EB was able to correctly estimate the position of the agent in space via the integration of sensory information. This process resulted from the weighed transformation of a flow of sensory inputs in a sequence of selections. By establishing a winner at each step in the competition among received inputs, the EB was then able to gate all but a single motor response. In turn, this clear-cut transition of activities in the LAL (Figure 5A) generated the visible spatial navigation of the simulated insect. Interestingly, in the complex maze arena, successful navigation correlated in several instances with periodic oscillations among EB modules (Figure 4). These oscillations were favored by the self-motion information conveyed by the body orientation units and premotor activity. Due to these inputs, the execution of turning behavior alters the incoming input and, because it is becoming part of the sensory input, is affecting the execution of future actions. This information loop resulted in oscillatory activity and cycles of motor sequences, thereby defining a cyclic attractor.

### Navigation with altered activity levels of EB layers and modules

To further explore the role of the EB in spatial navigation, we simulated either the deactivation or the over-activation of EB layers and modules, by manipulating the value of the constant *b*_*j*_ in equation 1. In the first set of manipulations, any decrease in the activity of EB layers resulted in a proportional decrease of the inhibitory gating function toward the LAL. The model was robust to low levels of manipulation (*b*_*j*_ = −0.5). In contrast, the more EB modules and layers were artificially impaired in their activity, the higher the chances of simultaneously co-activating multiple modules of the LAL (*b*_*j*_ < −0.5). This co-activation was interpreted in the model as the attempt to trigger multiple, conflicting motor outputs, such as “*turn left and right at the same time*” (cf. Figures 5A,B).

In the second set of manipulations, we artificially increased the baseline activity of EB layers and modules. This altered the signal to noise ratio in the entire CX, impairing the transient stability of stimulus-driven dynamics characterizing the EB under control conditions. At lower levels of over-activation (*b*_*j*_ = 0.5), the simulated insect required more time to change a selected behavior. This effect of over-activation is highlighted by a comparison we established for 50 (randomly selected) configurations of parameters that were found to be successful in reaching either of the two target areas in the open arena, whereby the simulated insect relied on all sensory input sources. These successful configurations were tested again after over-activating one layer in the EB. The comparison showed a significant increase in the simulated time required to reach the target area [4.9 ± 2.3 seconds vs. 3.6 ± 1.3 seconds, *p* < 0.001, *t*_(49)_ = −3.58]. Finally, at high levels of over-activation affecting all EB layers (0.5 < *b*_*j*_ < 1.0), the ability of the simulated insect to perform any selection and change it in response to new incoming stimuli was compromised, resulting in irresponsive and unsuccessful navigation behavior (cf. Figures 5A,C).

### Intentional spatial navigation

Under this condition, we tested whether the connectome of the CX, as defined in our model, could account for the flexible selection among different courses of action and navigation paths (e.g., Jourjine et al., 2016). Depending on interoceptive signals (e.g., hunger or thirst), the simulated insect had to decide which of two known repertoires to recall. Each repertoire putatively allowed to reach one of the two desired outcomes (e.g., food or water), each located in a different target area. In comparison with the tests simulating sensory-driven navigation, under this condition, a configuration of parameters or behavioral phenotype was considered successful if it allowed reaching both target area, in a sequence determined by the desired outcome. The search algorithm found a ratio of 90 solutions per million behavioral phenotypes, under the condition of open arena. The simple and complex maze condition provided one and zero solutions per million attempts, respectively (see also Supplementary Videos 7, 8).

## Discussion

Previous studies identified the central complex as a key neural correlate involved in processing sensory guidance cues and mediating behavioral outputs that together orchestrate spatial navigation in insects (Strausfeld and Hirth, 2013; Pfeiffer and Homberg, 2014; Turner-Evans and Jayaraman, 2016; Webb and Wystrach, 2016). However, the neural mechanisms underlying sensory integration and motor action selections have remained largely elusive. In particular, it is not yet understood how the CX exploits sensory inputs, including internal representations of head-body orientation, to realize motor functions associated with spatial navigation. Here we presented an *in silico* interrogation of the computational role the CX can play in sensory integration for motor action selection and spatial navigation. A simulated insect was tasked to navigate a series of environments of increasing complexity, in order to reach either one of two or both target areas in 2-dimensional arenas, whilst avoiding obstacles. The CX, which orchestrated changes in orientation and forward movements of the simulated insect, was characterized by a bio-constrained neural connectome (Fiore et al., 2015; Wolff et al., 2015; Kottler et al., 2017). The simulated insect relied on this structure to process a variety of sensory inputs, store navigation strategies and recall them as either a response to the stimuli perceived or depending on desired outcomes.

The simulations show our model of the CX is compatible with the sensory-driven multi-stable dynamics that characterize the presence of multiple attractor states (Fiore et al., 2015). This key computational feature has been formally described as an essential requirement for action selection across species, as these dynamics allow a neural system to perform a transient winner-take-all competition (Rabinovich et al., 2001; Afraimovich et al., 2008). Such competition, established among weighed sensory inputs, grants the suppression of noise and weak competitors, but does not prevent adaptation, so that the winning signal can change as a function of the sensory input. In the simulations, a continuous stream of sensory inputs is processed in our bio-constrained model of the CX to select sequences of actions resulting in spatial navigation. Furthermore, the simulations show this neural structure integrates multiple sensory sources, simulating how action selection and navigation can be guided by weighed information about polarized light, visual landmarks, view-based panoramas or self-motion and body orientation (Neuser et al., 2008; Ritzmann et al., 2008; Lin et al., 2014; Seelig and Jayaraman, 2015; Varga and Ritzmann, 2016; Omoto et al., 2017).

This process of sensory-driven motor selection is weighed by the columnar formation characterizing the CX, and in particular afferent EB connectivity. In the proposed model, the parameters regulating the weights of EB afferents and associated behavior are fixed and determine reactive responses. These weights could be either genetically determined, identifying innate motor behaviors, or they can be shaped by experience, as found in dopamine-dependent learning processes (Waddell, 2010, 2013; Lin et al., 2014). The latter case entails the insect would be able to form sensory-motor memories about stimulus-response associations, akin to habits.

Finally we tested whether our model could also account for a simplified form of intentional navigation that would overcome the limits posed by reactive sensory-driven navigation. In our test, we show the simulated CX could efficiently store two courses of actions in a single configuration of parameters or behavioral phenotype, each leading to a different target area. This feature allowed the model to arbitrate among different paths and select the sequence of actions that could satisfy a desired outcome. Our computational investigation suggests the CX can form rudimentary representations of space-related action-outcome contingencies and trigger the sequence of movements required to reach a desired position in space. Such a combined representation of motor and spatial information can be exploited to guide navigation and pursue a goal. We argue that the presence of this form of spatial memory can be an indication of a rudimentary mental representation of the environment (Tolman, 1932; Cheeseman et al., 2014).

In our simulations, we assumed that, to produce a simplified motor output, the neural system integrated different sources of information: (1) visual landmarks in the arena and their angular position; (2) body orientation and self-motion. The simulations show none of these input sources was sufficient, if considered alone, to guide the behavior of the simulated insect toward any of the target areas. Thus, the EB was required to integrate its multisensory inputs to generate a transient selection that would adapt to the continuous change of sensory information. The output of the EB in turn could “gate” the appropriate premotor response in the LAL, generating a sequence of actions in response to this multisensory stream of inputs. Importantly, self-localization in space can be accomplished also by relying on sensory integration of non-visual sources. For instance, antennal mechanosensations, which are also conveyed toward the EB (Ritzmann et al., 2008; Harley and Ritzmann, 2010; Guo and Ritzmann, 2013) can provide information about obstacles and landmarks, where surface features take the place of color or celestial e-vector orientations of polarized light (Heinze and Homberg, 2007). Therefore, these sensory inputs can be exploited also by nocturnal animals to trigger sequences of actions and guide successful spatial navigation (el Jundi et al., 2015).

This hypothesis, assuming multi-stable dynamics for the generation of sequences of actions, may seem at odds with recent findings, which suggest that the EB is characterized by ring attractor dynamics encoding an abstract internal representation of the fly's heading direction (Seelig and Jayaraman, 2015; Kim et al., 2017). Indeed, these studies show visual cues presented to head-fixed *Drosophila* result in a bump-like activity pattern largely confined to one EB module/wedge that can move along the EB and its wedges according to position changes (Seelig and Jayaraman, 2015; Kim et al., 2017). These dynamics have been interpreted as indicative of the presence of a ring attractor, which could be implemented either in specific layers of the EB (e.g., E-PG neurons as suggested by (Kim et al., 2017)) or in the entire neuropil. Different from multi-stable dynamics which are characterized by a finite set of separate stable states, in a ring attractor an infinite set of contiguous stable states respond in the continuum to changes in the input stimuli. These dynamics are reminiscent of those suggested for head direction cells in mammals (Taube, 2007), which encode present head and body direction. Despite compelling data showing how changes in orientation toward visual stimuli are encoded in the EB, the dynamics reported in these studies (Seelig and Jayaraman, 2015; Kim et al., 2017) have also highlighted the presence of clear discontinuity in the state transitions, as a function of the stimulus position (Kim et al., 2017–see their video s9 and related model comparison discussion).

We argue, a different interpretation of these data is supported by both our model and recent computational (Kakaria and de Bivort, 2017; Varga et al., 2017) and functional analyses (Omoto et al., 2017). This interpretation suggests information about body orientation is encoded upstream of the EB wedges and propagated toward the EB as one of several sensory inputs. Importantly, our interpretation does not limit the EB to the representation of head and body orientation, as the ring attractor hypothesis might suggest. The problem with such limitation of EB functions is that the gating exerted on the premotor area of the LAL would be guided by ring attractor dynamics, and would be reduced to the execution of changes of head and body orientation. Such a conclusion would conflict with data showing the important role of the EB in a wide range of motor and cognitive responses to diverse sensory stimuli, including e.g., flying (Weir and Dickinson, 2015), walking (Strauss and Heisenberg, 1993; Kottler et al., 2017), or place learning (Ofstad et al., 2011). All these functions are aided by the presence of a head direction information, as changes of position in space generally take into account the actual body orientation. Nonetheless, these motor and memory functions do not require, nor are limited to, the representation of head and body orientation, as an agent can perform multiple actions (walking, flying, grooming etc.) independently of its body orientation.

Despite the inclusion of several known features characterizing the local connectome of the CX, we acknowledge the presence of important limitations in the proposed model. Several aspects of the neural organization of the EB have not been described in the literature, yet. Details regarding those aspects of the model that come from biological data as stated in the literature as well as limitations in that data set that caused us to make reasonable assumptions can be found in the Methods section. For instance, a plausible hierarchy among EB layers (Fiore et al., 2015) would configure pathways of information processing that would affect the computational functions of the entire CX. Thus, further developments of the model will be necessary to include the micro-organization of the internal structures of the CX. Nonetheless, the dynamics characterizing our model rely on the macro organization of the connectivity among internal structures of the CX and suggest computational roles that account for a wide variety of data and behaviors. In particular, it is the combined effect of columnar input organization and lateral inhibitions that results in the hypothesized transient winner-take-all competitions essential for action selections and their assembly into action sequences. Furthermore, columnar connectivity targeting distinct modules in EB and LAL and associated multi-stability may indicate a hierarchical organization, akin the functional anatomy of the vertebrate basal ganglia (Fiore et al., 2015). In vertebrates, information about head and body orientation is found in the striatum as part of multiple sensory inputs that are computed in this nexus of the basal ganglia (Taube, 2007; Kim et al., 2014; Barter et al., 2015).

Our findings reveal that depleted activity in multiple EB layers and wedges cause conflicting motor output, whereas simultaneous co-activation of multiple EB layers and wedges result in random and ultimately unsuccessful spatial navigation. In the first case, too many conflicting motor commands are selected (*you can't turn left and right at the same time*), and in the second case no motor command is selected, thus causing inaction comparable to indecision or lack of motivation. In both cases, the net result *inaction* is caused by impaired action selection which in turn affects spatial navigation. Our computational data suggest that the functional nexus between wedge-specific columnar PB-EB (Green et al., 2017; Turner-Evans et al., 2017) and EB-LAL (Fiore et al., 2015) feedback loops, together with inhibitory activity from EB ring neurons (Kottler et al., 2017), code for neural mechanisms underlying sensory integration and motor action selection for spatial navigation. This hypothesis is supported by recent studies in *Drosophila* (Green et al., 2017; Turner-Evans et al., 2017) and cockroaches (Martin et al., 2015; Varga and Ritzmann, 2016) and leads to testable predictions, for example that targeted layer and/or wedge-specific activity manipulations of the EB in *Drosophila* affect goal-directed behavior like turning. It will be interesting to see the outcome of such experiments.

## Author contributions

VF and FH conceived and designed the project; BK provided graphical representation of CX; VF performed experiments; VF, BK, XG, and FH analyzed the data. VF and FH prepared the manuscript on which the authors commented.

### Conflict of interest statement

BK is co-founder of BFKLab LTD. The other authors declare that the research was conducted in the absence of any commercial or financial relationships that could be construed as a potential conflict of interest.

## Supplementary material

The videos show examples of simulated successful navigation, under different conditions. In each run, the starting location of the agent is selected randomly within the 100 locations in the South-West sector 1 of the arena. Target locations are found in sectors 14 (Target 1) or 16 (Target 2); the navigation is successful if the agent reaches its designated target within 30 s of simulated time.

Supplementary Video 1 Agent reaching Target 1 under the condition of open arena.

Supplementary Video 2 Agent reaching Target 2 under the condition of open arena.

Supplementary Video 3 An example of successful navigation toward Target 1 under the condition of simple maze.

Supplementary Video 4 An example of successful navigation toward Target 2 under the condition of simple maze.

Supplementary Video 5 The behavior of the simulated agent under the condition of complex maze, as it successfully reaches Target 1.

Supplementary Video 6 The behavior of the simulated agent under the condition of complex maze, as it successfully reaches Target 2.

Supplementary Video 7 An example of “intentional spatial navigation”, defined as the ability of a single phenotype to discriminate between putative goals and to pursue the appropriate course of action. The simulated agent is shown in two consecutive runs as a different goal is activated in each run. The order of the goals is fixed (e.g., hunger first and thirst second) so that the simulated agent is required to pursue Target 1 in the first run and Target 2 in the second run (e.g., food first and water second). The video shows the simulated agent reaching its target sectors in the order defined by its active goals, under the condition of open arena.

Supplementary Video 8 An example of “intentional spatial navigation,” defined as the ability of a single phenotype to discriminate between putative goals and to pursue the appropriate course of action. The simulated agent is shown in two consecutive runs as a different goal is activated in each run. The order of the goals is fixed (e.g., hunger first and thirst second) so that the simulated agent is required to pursue Target 1 in the first run and Target 2 in the second run (e.g., food first and water second). The video shows the simulated agent reaching its target sectors in the order defined by its active goals, under the condition of simple maze.
